# Impact of socioeconomic status and subjective social class on overall and health-related quality of life

**DOI:** 10.1186/s12889-015-2014-9

**Published:** 2015-08-15

**Authors:** Jae-Hyun Kim, Eun-Cheol Park

**Affiliations:** Department of Public Health, Graduate School, Yonsei University, Seoul, Republic of Korea; Institute of Health Services Research, Yonsei University, Seoul, Republic of Korea; Department of Preventive Medicine, Yonsei University College of Medicine, Seodaemun-gu, Seoul Republic of Korea

**Keywords:** Quality of life, Socioeconomic status, Health-related quality of life

## Abstract

**Background:**

Our objective was to investigate the impact of socioeconomic status and subjective social class on health-related quality of life (HRQOL) vs. overall quality of life (QOL).

**Methods:**

We performed a longitudinal analysis using data regarding 8250 individuals drawn from the Korean Longitudinal Study of Aging (KLoSA). We analyzed differences between HRQOL and QOL in individuals of various socioeconomic strata (high, middle, or low household income and education levels) and subjective social classes (high, middle, or low) at baseline (2009).

**Results:**

Individuals with low household incomes and of low subjective social class had the highest probability of reporting discrepant HRQOL and QOL scores (B: 4.796; *P* < 0.0001), whereas individuals with high household incomes and high subjective social class had the lowest probability of discrepant HRQOL and QOL scores (B: −3.625; *P* = 0.000). Similar trends were seen when education was used as a proxy for socioeconomic status.

**Conclusion:**

In conclusion, both household income/subjective social class and education/subjective social class were found to have an impact on the degree of divergence between QOL and HRQOL. Therefore, in designing interventions, socioeconomic inequalities should be taken into account through the use of multi-dimensional measurement tools.

## Background

Differences in socioeconomic status (SES), as assessed by income or educational achievement, are associated with large disparities in health status [1]. In Western European countries and in the U.S, the association between SES and health follows a common pattern [2–4]: the lower individuals are with respect to SES, the poorer their state of health. Similar results have been obtained in different countries, irrespective of cultural background or economic growth [5]. The association between SES and health outcomes persists across the life cycle [6] and across multiple measures of health, including health status [7], morbidity [8], mortality [9], self-assessed health [3, 10], and disease prevalence [11].

Many investigators find it difficult to ascertain which measures of SES are valid, which can be applied to multiple outcomes, and which are most relevant for specific conditions. In addition, while most researchers understand they must control for the effects of SES when analyzing health outcomes, many regard different measures of SES as interchangeable. In reality, education and income capture different aspects of social position and, thus, have distinctive characteristics [12]. For example, income is sensitive to changes in life circumstances and time, resulting in age limitations. Meanwhile, education is known to influence one’s ability to make informed decisions [13]. For example, previous research indicates that a higher level of education is associated with increased understanding of prostate health [14] and reduced pelvic function distress [15].

Disparities in health outcomes may stem from discordant social comparisons [16–18]; in wealthier countries, it has been argued, it is not economic level per se, but the distribution of income and wealth that is most important for the health status of a population [19]. Previous research suggests that subjective social class most accurately captures subtle aspects of social status, as it taps into psychosocial processes influenced by one’s social context [20]. Subjective social class has been found to better predict current physical and mental health (i.e., depression, negative affect, pessimism, reported stress, and general health ratings [17, 21]), and even mortality [17, 22].

In the last two decades, as populations have aged and the burden of chronic diseases has grown heavier, researchers have paid increasing attention to both quality of life (QOL) and health-related quality of life (HRQOL). Previous research [23] indicates that an individual’s subjective perception of their physical health, psychological health, social functioning, and environment, or “subjective quality of life”, is an independent determinant of wellness and disease burden. SES measures, such as income and education, have the potential to impact both HRQOL and QOL [24], and they have also been found to influence an individual’s life opportunities [25, 26]. Life opportunities can manifest in various ways, such as in the availability of healthcare resources or in an individual’s perception of his or her HRQOL, defined as the impact of a specific illness, injury, medical treatment, or health services policy on QOL [27]. QOL is used to describe a person’s general well-being. The World Health Organization QOL Group defines QOL [28] as: “Individuals’ perception of their position in life in the context of the culture and the value systems in which they live in relation to their goals, expectations, standards and concerns.” It is a broad concept, reflecting the fact that QOL may be affected in a complex manner by individuals’ physical health, psychological state, level of independence and social relationships, all of which represent salient features of the environment. Thus, the concept of HRQOL is often used to assess the impact of illness on QOL [29].

A previous cross-sectional study [30] investigated whether quality of life (BREAST-Q) and health-related quality of life (RAND 36) were directly influenced by surgery. However, no studies have investigated which of these two variables (QOL or HRQOL) is more heavily influenced by socioeconomic variables. Therefore, the primary aim of this study was to investigate whether discordant socioeconomic status and subjective social class were associated with discrepancies between HRQOL and QOL scores.

## Methods

### Study sample & design

Data for this study were drawn from the Korean Longitudinal Study of Aging (KLoSA), a nationwide multistage, stratified, cluster sampling survey of Koreans dwelling in the community. KLoSA is conducted by the Korea Labor Institute to collect the basic data needed to devise and implement effective social and economic policies that address emerging trends related to population aging. The original KLoSA study population comprised South Koreans aged 45 years or older in 2006 who lived in 15 large administrative areas. The survey is repeated every even-numbered year.

In the baseline survey in 2006, 10,254 individuals in 6171 households (1.7 individuals per household) were interviewed using a computer-assisted personal interviewing method. The second survey, in 2008, followed up with 8688 subjects, representing 86.6 % of the original panel. The third survey, in 2010, followed up with 7920 subjects, representing 80.3 % of the original panel. Finally, the fourth survey, in 2012, followed up with 7486 subjects, representing 76.2 % of the original panel. We used only 2008 and 2010 data, which included a household income question targeted at adults over the age of 45.

Of these participants, we excluded 435 subjects with no information on our variables of interest (household income, education, and subjective social class) for 2008. In addition, we excluded two subjects with no QOL data for 2008, and one subject with no data of any kind in 2008. We also excluded nine subjects with no information on our variables of interest (household income, education, and subjective social class) for 2010. In addition, we excluded two subjects with no QOL data for 2010, and two subjects with no data at all for 2010.

Korean Longitudinal Study of Aging (KLoSA) data are available in a national public database (website: http://www.kli.re.kr/klosa/en/about/introduce.jsp) and thus, ethical approval to conduct the study is not needed.

### Independent variables

#### Household income

Household income was divided into tertiles using the SAS rank function, from lowest (1) to highest (3).

#### Education level

Education was categorized into three groups: (1) middle school or lower, (2) high school, and (3) college or higher.

#### Subjective social class

Subjective social classes were determined by asking the respondents to assess their social class and rank themselves. The items were coded so that a higher score indicated a higher subjective social class. The item was rated 1 (Low-Low) to 6 (High-High). The response “Low-High” or “Low-Low” indicated “Low,” “Middle-Low” or “Middle-High” indicated “Middle,” and “High-High” or “High-Low” indicated “High.”

#### Gap between socioeconomic stratum and subjective social class

This gap represents the difference between household income [high, middle, low] and education [**≥**college, high school, **≤**middle school] and subjective social class [high, middle, low]. Therefore, it was categorized into nine groups for household income and nine for education: HL [high (**≥**college) - low], HM [high (**≥**college) - middle], HH [high (**≥**college) - high], ML [middle (high school) - low], MM [middle (high school) - middle], MH [middle (high school) - high], LL [low (**≤** middle school) - low], LM [low (**≤** middle school) - middle], and LH [low (**≤** middle school) - high].

### Dependent variables

#### HRQOL

We used the Euro-QoL visual analog scale (EQ VAS) to measure HRQOL. The HRQOL was measured as a response to the simple question “How do you usually perceive your health-related quality of life?” [31, 32]. This scale was specifically designed to capture overall health status. The HRQOL recorded the respondent’s current health state on a vertical, visual analog scale. The endpoints were labeled “best imaginable health state” (100) and “worst imaginable health state” (0). This information was used as a quantitative measure of health outcomes as judged by the individual respondents.

#### Subjective QOL

The subjective QOL was measured using the simple question “How is your overall quality of life?” and was a proxy indicator of the current health status of each respondent. Subjective QOL recorded each respondent’s current overall state on a vertical, visual analogue scale ranging from “best imaginable overall state” (100) to “worst imaginable overall state” (0). To assess QOL, an instrument measuring general well-being was used. The subjective QOL instruments included items addressing physical function, role-physical, bodily pain, general health, vitality, social function, role-emotional, and mental health.

#### Difference in HRQOL and QOL

We analyzed the difference between HRQOL and QOL as a dependent variable.

### Control variables

Age was divided into seven categories: ≤49, 50–54, 55–59, 60–64, 65–69, 70–74, and ≥75 years. Residential regions were categorized as urban (Seoul, Daejeon, Daegu, Busan, Incheon, Kwangju, and Ulsan) or rural (areas not classified as a city). Individuals were classified as currently married or not currently married, with the latter group including those previously married, widowed, or divorced. Employment status was divided into two categories: employed and unemployed (the latter category including housewives and students). Individuals were categorized as current users, former users, or never users of alcohol and cigarettes. Number of contact with friends was divided into five categories: every day, 1–2 times a week, 1–2 times a month, 3–6 times a year, and never. Self-rated health was assessed with the question “How do you usually perceive your health?” [33]. A response of “insufficient” or “very insufficient” was considered to indicate “Bad,” the response “normal,” was considered to indicate “Normal,” and a response of “sufficient,” or “very sufficient” was considered to indicate “Good.” The presence of self-reported depressive symptoms, categorized as “yes” or “no,” was extracted from the response to the question “Have you ever felt sadness or despair that hindered everyday life and continued for 2 weeks or more during the last year?” The number of chronic diseases (hypertension, diabetes, cancer, pulmonary disease, liver disease, coronary heart disease, cardiovascular disease, mental disease, and arthritis) was also included in our models, as were the number of offspring and year (time) of survey.

### Analytical approach and statistics

Analysis of variance (ANOVA) and generalized linear mixed models were used to investigate the effect of gap between socioeconomic stratum and subjective social class on difference HRQOL and QOL among old adults. For all analyses, the criterion for significance was P ≤ 0.05, two-tailed. All analyses were conducted using the SAS statistical software package version 9.2 (SAS Institute Inc., Cary, NC, USA).

### Generalized linear mixed effects model (SAS® Proc Glimmix)

Proc Glimmix is a generalized linear model procedure that permits the specification of a mixed multiple regression model. In a generalized linear mixed model, the observations of one individual over time are not independent of each other, and the model also takes into account the fact that the repeated observations of each individual are correlated. In all mixed models presented, only the intercept was allowed to vary between subjects, and regression slopes were assumed to be fixed effects; random intercept models were applied to our data. The random intercept variance is reported as σ^2^. To determine whether the probability of a difference between HRQOL and QOL changed over time, we included time (year of survey) in the model as a categorical covariate; the regression coefficient was used to estimate both the change in probability of a difference between HRQOL and QOL and the association with our independent variables, annually.

## Results

Table 1 presents the general characteristics of the 8250 research subjects at baseline as well as the association between each variable of interest and HRQOL, QOL, and the difference between HRQOL and QOL (Table 1).

**Table 1:** General characteristics of the 8,250 study subjects and association of each covariate with HRQOL, QOL, and the difference in HRQOL and QOL at baseline (2008)

	Total	Unweighted %	Weighted %	HRQOL			QOL			Difference in HRQOL and QOL		
				Weighted	Weighted	Weighted	Weighted	Weighted	Weighted	Weighted	Weighted	Weighted
				Mean	SD	*P*-value	Mean	SD	*P*-value	Mean	SD	*P*-value
Age (years)						<0.0001			<0.0001			<0.0001
≤49	1,407	17.1	11.9	44.2	805.8		54.0	727.4		-9.8	693.3	
50–54	1,160	14.1	9.7	49.7	783.3		56.7	699.7		-7.0	696.7	
55–59	1,252	15.2	14.0	53.7	892.3		59.3	778.5		-5.6	752.5	
60–64	1,140	13.8	16.9	56.3	990.2		61.9	858.4		-5.6	841.2	
65–69	1,159	14.1	17.0	59.7	944.0		63.4	848.7		-3.7	832.2	
70–74	1,296	15.7	18.4	62.3	904.0		63.0	815.7		-0.7	879.6	
≥75	836	10.1	12.1	64.0	905.7		63.7	831.4		0.3	889.1	
Sex						<0.0001			<0.0001			<0.0001
Male	3,616	43.8	47.4	59.5	946.2		62.2	818.0		-2.7	828.1	
Female	4,634	56.2	52.6	53.8	897.6		59.5	790.8		-5.8	784.7	
Residential region						<0.0001			<0.0001			<0.0001
Urban	3,667	44.5	45.4	56.7	942.2		59.1	845.1		-2.4	797.1	
Rural	4,583	55.6	54.6	56.3	915.0		62.2	766.0		-5.9	807.9	
Marital status						<0.0001			<0.0001			<0.0001
Married	6,459	78.3	81.1	58.5	907.1		62.7	770.5		-4.3	806.4	
Single	1,791	21.7	18.9	47.9	921.3		52.4	839.7		-4.5	807.5	
Employed												
Yes	3,555	43.1	48.8	62.4	857.8		63.8	778.8		-1.4	823.5	
No	4,695	56.9	51.2	50.8	919.5		57.9	807.2		-7.1	776.6	
Smoking status						<0.0001			<0.0001			<0.0001
Never	5,721	69.4	66.8	55.5	907.5		61.0	783.4		-5.5	778.5	
Former smoker	1,028	12.5	12.7	57.9	974.6		62.0	842.9		-4.1	834.6	
Smoker	1,501	18.2	20.6	58.7	957.7		59.4	853.7		-0.7	867.6	
Alcohol use						<0.0001			<0.0001			<0.0001
Yes	3,036	36.8	40.9	61.3	881.2		62.7	778.1		-1.4	814.9	
Former user	823	10.0	9.7	49.8	1,056.3		56.9	944.5		-7.1	891.8	
No	4,391	53.2	49.4	53.8	899.9		59.9	787.3		-6.1	771.0	
Number of contact with friend						<0.0001			<0.0001			<0.0001
Never	566	6.9	6.9	40.2	1,029.0		46.6	943.8		-6.4	916.7	
3–6 times a year	591	7.2	7.6	53.3	843.1		60.6	667.0		-7.3	762.8	
1–2 times a month	1,645	19.9	20.7	58.7	890.7		61.5	759.8		-2.8	814.6	
1–2 times a week	2,807	34.0	33.7	57.4	885.0		61.5	771.0		-4.1	787.8	
Every day	2,641	32.0	31.1	58.4	921.9		62.7	808.6		-4.3	801.0	
Self-rated health						<0.0001			<0.0001			<0.0001
Good	2,866	34.7	38.7	68.0	786.5		67.2	714.7		0.8	791.7	
Normal	2,987	36.2	35.3	57.5	712.6		61.0	726.2		-3.6	716.9	
Bad	2,397	29.1	25.9	37.9	831.9		50.8	833.4		-12.9	815.7	
Depressive symptoms						<0.0001			<0.0001			<0.0001
Yes	613	7.4	7.0	35.8	959.4		44.0	914.0		-8.2	943.0	
No	7,637	92.6	93.0	58.0	889.6		62.0	768.6		-4.0	793.3	
Number of chronic diseases						<0.0001			<0.0001			<0.0001
0	7,131	86.4	86.9	57.9	912.6		61.5	792.7		-3.6	798.1	
1	963	11.7	11.3	48.3	906.5		56.8	837.0		-8.6	823.6	
≥2	156	1.9	1.8	38.6	928.1		50.8	910.7		-12.2	871.3	
Number of offspring						<0.0001			<0.0001			<0.0001
0	205	2.5	2.7	46.0	1,058.7		44.9	956.5		1.1	851.3	
1	543	6.6	7.5	56.5	1,024.3		59.1	886.3		-2.5	929.0	
2	2,812	34.1	25.3	60.9	924.9		63.2	831.3		-2.3	824.3	
3	2,089	25.3	12.6	57.6	905.0		62.2	770.2		-4.7	796.4	
4	1,223	14.8	12.8	51.3	857.9		58.0	755.7		-6.7	738.6	
≥5	1,378	16.7	39.2	48.2	821.9		57.6	695.7		-9.5	737.2	
Total	8,250	100.0	100.0	56.5	927.2		60.8	804.8		-4.3	806.6	
Gap between income and subjective social class						<0.0001			<0.0001			<0.0001
HH [High-High]	176	2.1	2.3	73.1	785.9		78.9	564.8		-5.8	759.6	
HM [High-Mid]	1,986	24.1	27.5	66.0	783.1		69.7	615.1		-3.7	750.6	
HL [High-Low]	638	7.7	8.8	58.2	870.6		58.1	765.2		0.1	932.7	
MH [Mid-High]	45	0.6	0.5	59.0	875.7		73.0	748.7		-14.0	712.7	
MM [Mid-Mid]	1,253	15.2	15.2	60.9	837.1		66.9	643.4		-6.0	807.2	
ML [Mid-Low]	1,453	17.6	17.9	49.6	906.1		52.2	757.5		-2.6	879.9	
LH [Low-High]	45	0.6	0.4	61.3	892.4		70.2	723.1		-8.9	442.6	
LM [Low-Mid]	968	11.7	10.3	57.2	805.4		65.1	634.4		-7.9	740.5	
LL [Low-Low]	1,686	20.4	17.3	40.9	851.4		46.0	775.3		-5.2	777.9	
Gap between education and subjective social class						<0.0001			<0.0001			<0.0001
HH [High-High]	94	1.1	1.2	76.0	709.5		81.4	495.6		-5.4	629.2	
HM [High-Mid]	575	7.0	8.2	67.4	812.2		70.2	673.4		-2.8	718.7	
HL [High-Low]	134	1.6	1.9	55.5	977.7		52.5	843.0		3.0	1,040.5	
MH [Mid-High]	92	1.1	1.1	69.5	867.8		75.7	619.7		-6.2	755.5	
MM [Mid-Mid]	1,435	17.4	19.6	65.7	774.0		69.0	610.8		-3.3	745.8	
ML [Mid-Low]	669	8.1	9.3	54.0	932.4		54.3	835.9		-0.3	922.9	
LH [Low-High]	80	1.0	0.8	59.5	854.4		71.7	742.9		-12.2	741.1	
LM [Low-Mid]	2,197	26.6	25.1	59.2	821.7		66.6	631.0		-7.4	785.9	
LL [Low-Low]	2,974	36.1	32.7	45.7	893.6		49.9	771.8		-4.2	816.7	
Gap between income and subjective social class						<0.0001			<0.0001			<0.0001
Overestimation of socioeconomic stratum	1,058	12.8	54.1	59.3	905.1		62.0	781.4		-2.7	830.5	
Accurate	3,115	37.8	11.1	57.5	812.2		65.6	647.0		-8.1	730.4	
Underestimation of socioeconomic stratum	4,077	49.4	34.7	51.7	959.9		57.3	858.6		-5.6	788.7	
Gap between education and subjective social class						<0.0001			<0.0001			<0.0001
Overestimation of socioeconomic stratum	2,369	28.7	19.4	59.8	938.0		60.8	857.0		-1.0	860.2	
Accurate	4,503	54.6	27.0	59.6	828.7		67.1	639.8		-7.5	783.8	
Underestimation of socioeconomic stratum	1,378	16.7	53.6	53.7	956.4		57.6	832.0		-3.9	791.3	
Income						<0.0001			<0.0001			<0.0001
Low	2,699	32.7	28.0	47.2	894.4		53.4	816.3		-6.2	761.9	
Middle	2,751	33.4	33.5	54.8	907.7		59.1	777.5		-4.3	849.3	
High	2,800	33.9	38.5	64.7	822.9		67.6	697.7		-3.0	800.0	
Subjective Social Class						<0.0001			<0.0001			<0.0001
Low	3,777	45.8	44.0	47.9	917.0		50.9	789.6		-3.1	849.3	
Middle	4,207	51.0	52.9	62.9	818.9		68.0	633.2		-5.2	768.8	
High	266	3.2	3.2	69.5	854.3		76.9	640.7		-7.4	716.4	
Education						<0.0001			<0.0001			<0.0001
≤Middle school	5,251	63.7	58.6	51.7	906.7		57.3	794.6		-5.7	805.9	
High school	2,196	26.6	30.1	62.2	866.7		64.7	759.2		-2.5	807.0	
≥College	803	9.7	11.3	66.3	869.3		68.4	779.8		-2.1	779.7	
Total	8,250	100.0	100.0	56.5	927.2		60.8	804.8		-4.3	806.6	

Table 2 shows the effects of various variables of interest on the difference between HRQOL and QOL. The baseline weighted mean differences between yearly HRQOL and QOL among individuals whose household income matched their subjective social class was −5.8 for individuals with high incomes and of high social class, −6.0 for those with medium incomes and of medium social class, and −5.2 for those with low incomes and of low social class (Table 2). In addition, Table 2 includes a scale indicating the effects of overestimated, accurate, or underestimated socioeconomic status, as reflected in subjective assessments of social class. When an individual’s subjective social class was higher than his/her socioeconomic status, as measured by household income, the difference between HRQOL and QOL was −2.724 (*P* < 0.0001); when an individual’s subjective social class was higher than his/her socioeconomic status, as measured by education, the difference between the two measures was −2.553 (*P* < 0.0001).

**Table 2:** Adjusted effect of gap between household income or education and subjective social class on difference between HRQOL and QOL

	Difference between HRQOL and QOL									
	Household income					Education				
	Estimate	SE	95 % CI		*P*-value	Estimate	SE	95 % CI		*P*-value
Scale of estimation										
Overestimation of socioeconomic stratum	-2.724	0.455	-3.616	-1.832	<0.0001	-2.553	0.318	-3.176	-1.929	<0.0001
Accurate	ref					ref				
Underestimation of socioeconomic stratum	0.134	0.324	-0.500	0.768	0.678	0.612	0.373	-0.119	1.343	0.101
Age (years)										
≤49	ref					ref				
50–54	-0.201	0.572	-1.322	0.920	0.725	0.105	0.570	-1.013	1.223	0.854
55–59	-2.068	0.591	-3.225	-0.910	0.001	-1.587	0.589	-2.741	-0.433	0.007
60–64	-2.367	0.613	-3.569	-1.165	0.000	-2.079	0.611	-3.277	-0.881	0.001
65–69	-1.888	0.658	-3.177	-0.598	0.004	-1.868	0.656	-3.153	-0.582	0.004
70–74	-2.114	0.736	-3.556	-0.671	0.004	-2.447	0.735	-3.887	-1.007	0.001
≥75	-2.937	0.758	-4.423	-1.451	0.000	-3.159	0.756	-4.640	-1.678	<0.0001
Sex										
Male	0.421	0.419	-0.400	1.242	0.314	-0.327	0.414	-1.138	0.484	0.430
Female	ref					ref				
Residential region										
Urban	1.935	0.278	1.389	2.480	<0.0001	1.640	0.278	1.096	2.184	<0.0001
Rural	ref					ref				
Income (Education)										
Low	1.880	0.482	0.935	2.825	0.030	2.876	0.433	2.028	3.724	<0.0001
Middle	0.604	0.473	-0.324	1.532	0.292	2.579	0.347	1.899	3.259	<0.0001
High	ref					ref				
Marital status										
Married	-3.270	0.389	-4.032	-2.507	<0.0001	-2.442	0.397	-3.219	-1.664	<0.0001
Single	ref					ref				
Employed										
Yes	1.108	0.327	0.466	1.750	0.001	1.849	0.328	1.205	2.492	<0.0001
No	ref					ref				
Smoking status										
Never	-1.786	0.434	-2.637	-0.936	<0.0001	-1.870	0.432	-2.716	-1.024	<0.0001
Former smoker	-1.209	0.482	-2.154	-0.264	0.013	-1.160	0.481	-2.102	-0.217	0.016
Smoker	ref					ref				
Alcohol use										
Yes	0.926	0.348	0.245	1.608	0.008	1.072	0.347	0.392	1.752	0.002
Former user	-0.029	0.490	-0.989	0.931	0.953	0.010	0.489	-0.948	0.968	0.984
No	ref					ref				
Number of contact with friend										
Never	-0.056	0.596	-1.223	1.111	0.925	-0.220	0.594	-1.385	0.945	0.711
3–6 times a year	-0.632	0.526	-1.663	0.398	0.229	-0.657	0.525	-1.685	0.372	0.211
1–2 times a month	0.117	0.389	-0.645	0.879	0.764	0.224	0.388	-0.537	0.984	0.565
1–2 times a week	0.295	0.340	-0.370	0.961	0.384	0.277	0.339	-0.387	0.941	0.413
Every day	ref					ref				
Self-rated health										
Good	11.978	0.412	11.171	12.785	<0.0001	12.173	0.410	11.369	12.976	<0.0001
Normal	8.307	0.373	7.577	9.037	<0.0001	8.359	0.371	7.631	9.087	<0.0001
Bad	ref					ref				
Depressive symptoms										
Yes	-0.331	0.569	-1.446	0.785	0.562	-0.585	0.568	-1.699	0.528	0.303
No	ref					ref				
Number of chronic diseases										
0	1.854	1.088	-0.278	3.986	0.089	1.903	1.085	-0.224	4.029	0.080
1	0.860	1.141	-1.376	3.095	0.451	0.912	1.138	-1.318	3.143	0.423
≥2	ref					ref				
Number of offspring										
0	4.441	0.852	2.772	6.110	<0.0001	4.262	0.849	2.597	5.926	<0.0001
1	0.275	0.530	-0.764	1.314	0.605	-0.024	0.530	-1.062	1.014	0.964
2	ref					ref				
3	-0.048	0.360	-0.754	0.659	0.895	0.140	0.359	-0.564	0.845	0.697
4	-0.192	0.485	-1.143	0.759	0.693	0.190	0.483	-0.756	1.136	0.695
≥5	-1.305	0.531	-2.346	-0.263	0.016	-0.922	0.529	-1.957	0.114	0.085
Year of survey										
2008	-0.036	0.273	-0.570	0.498	0.894	-0.037	0.272	-0.570	0.496	0.892
2010	ref					ref				

**Fig. 1:**
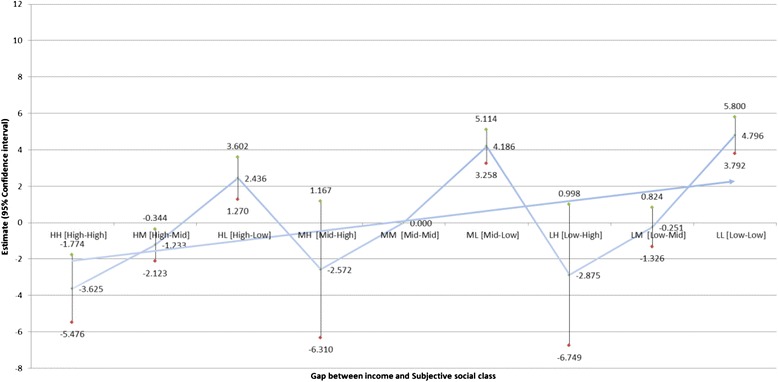
Adjusted effect of the gap between income and subjective social class on difference between HRQOL and QOL

**Fig. 2:**
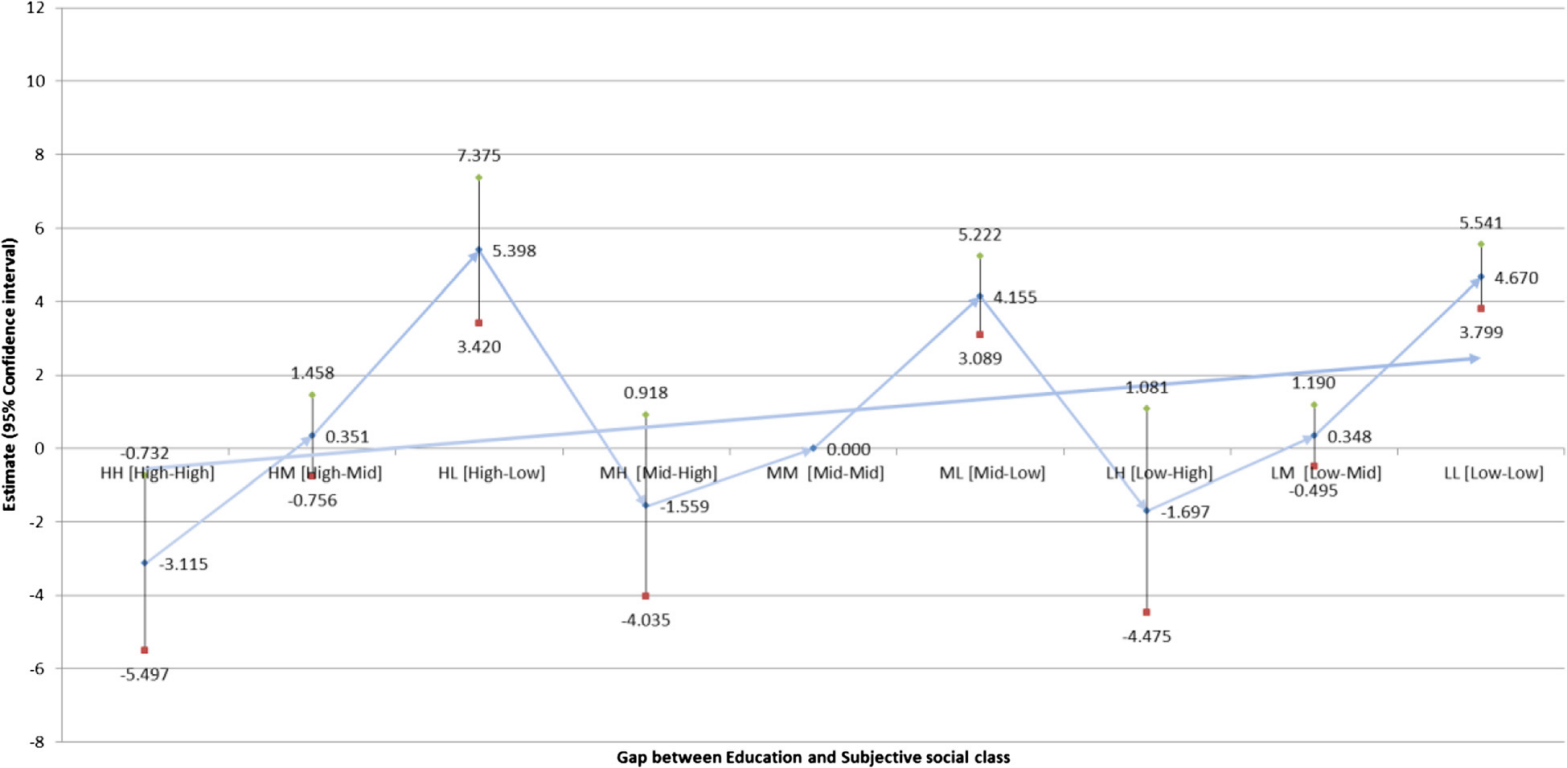
Adjusted effect of the gap between education level and subjective social class on difference between HRQOL and QOL

Table 3 indicates the impact of the gap between subjective social class and household income (Fig. 1) and education (Fig. 2) on the difference between HRQOL and QOL scores (Table 3). Individuals with low household incomes and of low subjective social class were the most likely to display a positive difference between HRQOL and QOL scores (B = 4.796; *P* < 0.0001), while those with high household incomes and of high subjective social class were the least likely to exhibit such a discrepancy (B = −3.625, *P* = 0.000). Similarly, individuals with a low education level and of low subjective social status were the most likely to exhibit a positive difference between HRQOL and QOL scores (B = 4.670; *P* < 0.0001), while individuals with a high education level and of high subjective social status were the least likely to do so (B = −3.115, 95 % CI: 0.568–0.862).

**Table 3:** Adjusted effect of gap between household income or education levels and subjective social class on difference between HRQOL and QOL

	Difference between HRQOL and QOL									
	Household income					Education				
	Estimate	SE	95 % CI		*P*-value	Estimate	SE	95 % CI		*P*-value
Gap between socioeconomic stratum and subjective social class										
HH [High-High]	-3.625	0.944	-5.476	-1.774	0.000	-3.115	1.215	-5.497	-0.732	0.011
HM [High-Mid]	-1.233	0.454	-2.123	-0.344	0.007	0.351	0.565	-0.756	1.458	0.534
HL [High-Low]	2.436	0.595	1.270	3.602	<0.0001	5.398	1.009	3.420	7.375	<.0001
MH [Mid-High]	-2.572	1.907	-6.310	1.167	0.178	-1.559	1.264	-4.035	0.918	0.218
MM [Mid-Mid]	ref					ref				
ML [Mid-Low]	4.186	0.473	3.258	5.114	<0.0001	4.155	0.544	3.089	5.222	<.0001
LH [Low-High]	-2.875	1.976	-6.749	0.998	0.146	-1.697	1.417	-4.475	1.081	0.231
LM [Low-Mid]	-0.251	0.549	-1.326	0.824	0.647	0.348	0.430	-0.495	1.190	0.419
LL [Low-Low]	4.796	0.512	3.792	5.800	<0.0001	4.670	0.445	3.799	5.541	<.0001
Age (years)										
≤49	ref					ref				
50–54	-0.026	0.568	-1.140	1.087	0.963	0.002	0.568	-1.112	1.115	0.998
55–59	-1.881	0.587	-3.031	-0.730	0.001	-1.832	0.587	-2.983	-0.681	0.002
60–64	-2.473	0.611	-3.670	-1.276	<0.0001	-2.422	0.611	-3.619	-1.225	<0.0001
65–69	-2.311	0.656	-3.596	-1.026	0.000	-2.260	0.656	-3.546	-0.975	0.001
70–74	-2.953	0.734	-4.391	-1.514	<0.0001	-2.897	0.734	-4.337	-1.458	<0.0001
≥75	-3.613	0.755	-5.093	-2.133	<0.0001	-3.563	0.756	-5.044	-2.082	<0.0001
Sex										
Male	0.304	0.416	-0.511	1.120	0.464	0.296	0.416	-0.520	1.111	0.477
Female	ref					ref				
Residential region										
Urban	1.716	0.277	1.174	2.259	<0.0001	1.700	0.277	1.157	2.242	<0.0001
Rural	ref					ref				
Income (Education)										
Low	0.057	0.499	-0.922	1.036	0.917	1.540	0.445	0.668	2.411	0.001
Middle	-0.330	0.478	-1.267	0.608	0.540	1.292	0.362	0.583	2.002	0.000
High	ref					ref				
Marital status										
Married	-2.179	0.396	-2.955	-1.404	<0.0001	-2.190	0.396	-2.965	-1.414	<0.0001
Single	ref					ref				
Employed										
Yes	1.504	0.328	0.861	2.147	<0.0001	1.510	0.328	0.867	2.153	<0.0001
No	ref					ref				
Smoking status										
Never	-1.355	0.432	-2.202	-0.509	0.002	-1.340	0.432	-2.187	-0.493	0.002
Former smoker	-0.848	0.480	-1.788	0.092	0.078	-0.817	0.480	-1.757	0.123	0.089
Smoker	ref					ref				
Alcohol use										
Yes	1.043	0.346	0.366	1.720	0.003	1.066	0.346	0.389	1.743	0.002
Former user	0.004	0.486	-0.949	0.958	0.993	0.006	0.486	-0.947	0.959	0.990
No	ref					ref				
Number of contact with friend										
Never	-0.607	0.592	-1.768	0.554	0.306	-0.618	0.593	-1.780	0.543	0.297
3-6 times a year	-0.625	0.522	-1.649	0.399	0.232	-0.628	0.523	-1.652	0.396	0.230
1-2 times a month	0.229	0.386	-0.528	0.986	0.553	0.224	0.386	-0.533	0.981	0.563
1-2 times a week	0.313	0.337	-0.348	0.974	0.354	0.329	0.337	-0.333	0.990	0.330
Every day	ref					ref				
Self-rated health										
Good	13.221	0.417	12.404	14.038	<0.0001	13.183	0.416	12.366	13.999	<0.0001
Normal	8.858	0.372	8.128	9.588	<0.0001	8.804	0.372	8.076	9.532	<0.0001
Bad	ref					ref				
Depressive symptoms										
Yes	-0.733	0.566	-1.842	0.376	0.196	-0.696	0.566	-1.805	0.412	0.219
No	ref					ref				
Number of chronic diseases										
0	1.948	1.080	-0.169	4.065	0.072	1.971	1.080	-0.146	4.088	0.068
1	1.038	1.133	-1.182	3.258	0.360	1.056	1.133	-1.164	3.277	0.351
≥2	ref					ref				
Number of offspring										
0	3.458	0.848	1.796	5.120	0.000	3.506	0.847	1.845	5.167	<0.0001
1	-0.171	0.527	-1.205	0.862	0.746	-0.174	0.527	-1.208	0.860	0.742
2	ref					ref				
3	0.003	0.358	-0.698	0.705	0.992	-0.018	0.358	-0.720	0.683	0.960
4	-0.216	0.482	-1.160	0.728	0.655	-0.228	0.482	-1.173	0.716	0.637
≥5	-1.394	0.528	-2.428	-0.360	0.010	-1.412	0.528	-2.446	-0.377	0.009
Year of survey										
2008	-0.136	0.271	-0.666	0.395	0.616	-0.132	0.271	-0.662	0.399	0.627
2010	ref					ref

In addition, we analyzed the associations between HRQOL and QOL discrepancies and socioeconomic status (as measured by household income and education) and subjective social class (Table 4). The adjusted effect of the gap between socioeconomic status and subjective social class on the difference between HRQOL and QOL increased across the socioeconomic spectrum, but similar findings were not found with respect to household income.

## Discussion

In this study, our primary purpose was to investigate the impact of disadvantaged socioeconomic status and subjective social class on QOL measurement tools (QOL and HRQOL). Furthermore, we focused on determining which dependent variable (HRQOL or QOL) is more heavily influenced by socioeconomic variables.

To do so, we conducted a longitudinal assessment of a nationally representative sample of adults aged 45 years and higher in South Korea.

Overall, the variation in QOL observed in relation to SES was greater than the variation observed in HRQOL (Appendix 1 and 2), but subjective social class was more strongly associated with both HRQOL and QOL than either income or education (Appendix 3).

We found that gaps between income and subjective social class were associated with increased differences between HRQOL and QOL, as were gaps between education and subjective social class. The QOL scores of individuals of high subjective social class tended to be higher than their HRQOL scores, whereas the HRQOL scores of individuals of low subjective social class tended to be higher than their QOL scores. This trend was observed at all levels of household income and education. That is, for a given household income, the probability that HRQOL was higher than QOL increased as subjective social class decreased. Similarly, the probability of HRQOL being higher than QOL was elevated in individuals with low household income (or low education) and of low subjective social class. Our results indicated that within any socioeconomic stratum, those of low subjective social class tended to exhibit a greater difference between HRQOL and QOL scores.

These associations were independent of sociodemographic variables (age, sex, residential region, income, education, marital status, and employment status), health risks and behavioral variables (amount of contact with friends, smoking status, and alcohol use), health status (self-rated health, depressive symptoms, and number of chronic diseases), number of offspring, and the year of the survey.

Research has suggested that patients suffering from depression exhibit similar or worse subjective QOL scores than patients with chronic medical conditions or other severe mental illnesses (e.g., schizophrenia or bipolar disorder) [23]. Furthermore, researchers have found that effective treatments for depression do not always improve subjective QOL [34, 35]. It is generally agreed that subjective QOL is a construct determined by multiple factors [36]. Researchers have suggested that various clinical, sociodemographic, or biological factors may moderate subjective QOL and that value systems and cultural factors in particular may have a strong influence.

To date, there have been few studies examining the relationship between SES and HRQOL in Asian countries. Lam et al. examined the effect of HRQOL on health service utilitilization and validated the SF12 Health Survey in a Chinese sample [37]. In addition, several recent studies have examined SES and HRQOL, but all have focused on special populations such as the elderly living alone [38], the elderly with hearing impairments [39], or patients with diseases [40]. Meanwhile, studies regarding QOL and its relation to sociodemographic characteristics in South Korea have yielded inconsistent findings [41, 42]. Suh [42] found that education, income, employment status, and stage of disease impacted QOL, whereas age, religion, marital status, and type of adjunct therapy do not. However, Shim and Park [41] reported that “being religious” was the only sociodemographic characteristic that influenced QOL; age, education, employment status, income, time since operation, and number of chemotherapy rounds did not.

QOL, as defined by the World Health Organization (WHO), is a multidimensional construct with numerous physical, psychological, social, and economic components [28]. Despite the uncertainty inherent in the definition, HRQOL is a construct of high clinical relevance, as recent research has shown that it is an important predictor of other health outcomes [43]. Therefore, it has become increasingly important to understand the socioeconomic factors that influence QOL, including HRQOL [44], and our findings support previous research on this subject.

Our study’s purpose was to explore two separate socioeconomic measures and their effects on differences between HRQOL and overall QOL. In conclusion, the combination of high income-based status and subjective social class was found to have a greater impact on the difference between HRQOL and QOL scores (when comparing between HH and LL groups) than the combination of high education-based status and subjective social class. In addition, the combinations of household income/subjective social class and education/subjective social classes were both found to have an impact on the difference between QOL and HRQOL. Further studies are needed to test our hypothesis by developing precise socioeconomic measures (income, education and subjective social class) and determining their predictive value with respect to QOL or HRQOL.

Our study has a number of strengths and limitations. A major strength is that the participants in the survey are representative of the overall South Korean adult population and, because the sample size is large, our results can be generalized to the national level. Nevertheless, the potential for bias exists. For example, current household income may not adequately represent the standard of living for retired individuals, because it may not reflect all financial resources available; in addition, it disregards the cumulative effects of a lifetime of deprivation or privilege. Moreover, because current income may be a product of recent health, associations between income and health are subject to reverse-causation problems. A second problem is that respondents’ reports of their social class, depressive symptoms, QOL, and HRQOL are subjective and imperfect measures that may be affected by false consciousness or adaptation to resources. Third, because personality characteristics are likely to be associated with both subjective social class and QOL, including HRQOL, failure to include them in statistical models may lead to a distortion of the results and an exaggeration of observed effects. In addition to the above potential biases, which are likely to inflate the associations between subjective social class and some health variables, we recognize that our estimates may understate the potential effects of all factors on the difference between HRQOL and QOL due to the short follow-up period in our analysis. Fourth, although we analyzed longitudinal data, the results may reflect a bidirectional relationship in the association between the difference between HRQOL and QOL and disadvantaged socioeconomic status and subjective social class.

## Conclusions

According to our results, combined measures of household income/subjective social class and education/subjective social classes were both found to have an impact on the difference between QOL and HRQOL. Therefore, socioeconomic inequalities should be taken into account when designing interventions focusing on psychosocial factors. Our study provides additional evidence that gaps between socioeconomic status and perceived position in the social hierarchy may have important health implications with regard to the difference between HRQOL and QOL.

## Appendix 1

ᅟ

## Appendix 2

ᅟ

## Appendix 3

ᅟ
